# Impact of Novel Tools for Blended and Competency‐Based Learning in Veterinary Compounding and Prescribing

**DOI:** 10.1111/jvp.70062

**Published:** 2026-03-09

**Authors:** Sonja Bröer, Viviane Filor, Wolfgang Bäumer

**Affiliations:** ^1^ Institute of Pharmacology and Toxicology, School of Veterinary Medicine Freie Universität Berlin Berlin Germany

**Keywords:** active learning, compounding, first‐day skills, flipped classroom, prescribing

## Abstract

According to the European Association of Establishments for Veterinary Education (EAEVE), a graduate of veterinary medicine should be able to prescribe and dispense “medicines correctly and responsibly in accordance with legislation and latest guidance” (2019). In Germany, veterinarians can dispense drugs from their in‐practice pharmacy, which makes dispensing and compounding a higher educational priority than in other EU member states. This report describes recent efforts to improve the teaching of relevant first‐day skills in compounding and prescribing through active learning approaches such as blended learning and online learning resources, including flipped classroom settings and cloud‐based documents to practice prescribing at one of the German Schools of Veterinary Medicine. We assessed how the introduction of blended and case‐based learning and quizzes impacted learning outcomes and grades over the last 5 years, from 2019 to 2023. This timeframe included regular pre‐pandemic teaching operations, remote teaching during the pandemic, and a return to in‐person classes. Pandemic online teaching did not lead to worse outcomes in practical or theoretical knowledge; online tutorials for compounding improved student performance significantly during the pandemic, though performance declined after returning to in‐person teaching until quiz‐based extra points were introduced in 2023. Doubling prescription seminar time did not improve outcomes in 2020, but introducing a cloud‐based prescription exercise in 2021 led to better results, and overall grades in pharmaceutical and narcotics law have significantly improved since 2020. Novel tools for blended and case‐based learning improved all outcomes significantly and were positively evaluated by the students.

## 1 Introduction

Good knowledge of pharmacology and toxicology is critical for treating patients. A systematic review of human health care has revealed that 1 out of 30 patients are potentially harmed by preventable medication errors, with the majority being allocated to prescribing errors (Hodkinson et al. 2020). Most prescribing errors occurred in older, more severely ill patients with polypharmacy, but also could be related to poor storage (e.g., similar looking drug packaging), and dispensing practices (Hodkinson et al. 2020). While those mistakes are also likely to occur in veterinary clinical practice, less data is available. However, prescription errors were more prevalent than medication preparation or administration errors in a study in a large veterinary teaching hospital in North Carolina (Hepworth‐Warren et al. 2024). According to the European Association of Establishments for Veterinary Education (EAEVE), the “Day One Competences” that a veterinarian should have acquired in pharmacology at graduation include prescribing and dispensing “medicines correctly and responsibly in accordance with legislation and latest guidance” (2019). The EAEVE defines competences as a “concept that integrates knowledge, skills and attitudes. Competence requires acquisition of technical skills but further involves applying relevant knowledge, and having the confidence and ability to transfer what has been learnt to a variety of contexts. […] “Day One Competence” is the minimum standard required and is the starting point for a variety of roles in the veterinary profession (e.g., as Practitioner, Hygienist, Scientist, National Veterinary Services Officer, Animal Welfare Officer, Designated Veterinarian,…). […] Veterinary educational establishments are responsible for developing the day one competence of their students and ensuring that they have met the competences by the time they graduate” (2019). Apart from avoiding harm and choosing the appropriate treatment for the patient based on pharmacology and toxicology concepts, correct prescribing also requires knowledge in many other areas of the veterinary profession, such as basic science and modern public health challenges, including safe livestock and food production and antibiotic resistance.

In Germany, veterinarians can dispense drugs from their in‐practice pharmacy (§44 Tierarzneimittelgesetz, TAMG 2022a). Direct drug dispensing is also prevalent in other countries, such as Austria, Denmark, Latvia, Switzerland, and New Zealand. Thus, it is imperative to know the latest legislation on buying, importing, storing, prescribing, and dispensing veterinary medicinal products, controlled substances, and medicated feed. Veterinarians also need to know rules on the use of medicinal products outside the terms of the marketing authorization (referred to as “off‐label use” in this manuscript) across different species. Another legal option available to veterinarians in the European Union is compounding medicines for individual patients. This refers to the extemporaneous preparation of a medicinal product in accordance with a veterinary prescription when neither an authorized veterinary medicinal product (VMP), nor a medicinal product authorized for human use is available (according to Article 112–114, Regulation (EU) 2019/6). Due to the relevance and the interdisciplinary nature of pharmacology and medicine regulations across all veterinary sectors and human medicine, teaching the content merely in theory is insufficient. There is very little data on teaching practices and outcomes in veterinary pharmacology. A recent cross‐sectional analysis conducted across 17 medical schools in 15 European countries has evaluated the clinical pharmacology and therapeutics competencies of 895 final‐year medical students (Brinkman et al. 2017): The lowest level of knowledge was observed in drug interactions and contraindications, while the most errors were made in prescribing. More than 50% of prescriptions contained one or more errors, with the most prevalent errors being “less effective drug choice” (19.6%), “incomplete/incorrect drug prescription” (18.0%), and “overdosing” (17.9%) (Brinkman et al. 2017). These findings clearly indicate that not only is theoretical knowledge about drug selection and effects insufficient, but there is also a lack of technical proficiency in writing formally correct prescriptions. Furthermore, the students also reported that they did not feel confident in their prescribing skills, and almost half of them reported to have never written out a prescription during their studies (Brinkman et al. 2017). This is particularly concerning, because other studies have shown that recent graduates are writing the majority of prescriptions in hospital settings (cf. Dornan et al. 2009). As a follow‐up study, Brinkman et al. (2021) performed a direct comparison between traditional learning approaches and problem‐based learning concepts: They found that problem‐based learning improved the knowledge, prescribing skills, and confidence of the students significantly. These findings from human medicine and associated pharmacology teaching prompted us to think about making improvements in teaching relevant first‐day skills in compounding and prescribing in veterinary pharmacology education. We evaluated the impact of implementing blended learning, case‐based approaches, and quizzes on student performance and learning outcomes over a five‐year period (2019–2023), encompassing pre‐pandemic in‐person teaching, remote instruction during the pandemic, and the subsequent return to on‐campus learning. The use of digital media and offers for independent learning were evaluated by the students in questionnaires.

## 2 Materials and Methods

### 2.1 Subject

#### 2.1.1 Pharmaceutical and Narcotics Law

We teach national and EU regulations on veterinary medicinal products, medicated feed, and controlled substances for veterinary students in their 4th out of 6 years of veterinary school, after they have successfully passed their exam in pharmacology. According to the German regulation on veterinary licensing (Verordnung zur Approbation von Tierärztinnen und Tierärzten (TAppV) 2006), students have to prove that they can select and prescribe suitable medicines for at least three clinical cases and know the principles of determining maximum residue levels and deriving withdrawal periods. They must also compound two medications according to a prescription and charge them following the regulations applicable to medicine prices. In addition, students must demonstrate their knowledge of the relevant legal regulations regarding the traffic of medicinal products and narcotics and the regulations and measures to prevent residues in foods of animal origin. The above‐mentioned national regulation on veterinary licensing (TAppV 2006) is continuously updated to accommodate EU‐wide regulations for compounding and prescription of veterinary medicinal products. Thus, the subject “Pharmaceutical and narcotics law” includes three modules: a lecture, a practical compounding course, and a prescriptions seminar (Table 1). Consequently, the “Pharmaceutical and narcotics law” exam consists of three parts: (1) a practical compounding exam, which accounts for 20% of the final grade, (2) a written prescription exam, which also accounts for 20% of the final grade, and (3) an oral exam, consisting of another oral prescription for a specific veterinary indication and a theoretical question on legislation that account for the remaining 60% of the total grade. In total, veterinary students must fulfill at least 126 h of pharmacology and toxicology, including clinical pharmacology, pharmaceutical and narcotics law, drug prescription and compounding theory, residue formation and avoidance, and risk assessment.

**TABLE 1:** Differences in in‐person and online teaching hours, and teaching methods across the three parts of the subject “drug and narcotics law” between 2019 and 2023.

	Year	Hours	In‐person	Online	Obligatory attendance	Specials	Quizzes
Lecture	2019	18	18	—	No	Visits from external specialists	
	2020	18	—	18	No		x
	2021	18	7.5	11.5	No		x
	2022	18	18	—	No		x
	2023	18	18	—	No		x
Compounding course	2019	7.5	7.5	—	Yes		
	2020	7.5	1.5	6	Yes	Videos on compounding	
	2021	7.5	3	4.5	Yes		
	2022	6	6	—	Yes		
	2023	6	6	—	Yes		x
Prescription seminar	2019	1.5	1.5	—	Yes	Cases for self‐study	
	2020	3	—	3	Yes	Cases + meeting to discuss	
	2021	3	—	3	Yes	Cases, meeting + cloud‐based document	
	2022	3	—	3	Yes		
	2023	3	—	3	Yes		

### 2.2 Lecture

The lecture consisted of 18 h (1.5 h/week, Table 1). Pre‐pandemic lectures were traditional PowerPoint‐based presentations. In 2020 and most of 2021, the lecture was transferred to an online teaching format, with live and on‐demand sessions. In 2022, the faculty and students returned to in‐person teaching. Quizzes were performed regularly throughout in‐person and online teaching by an internal quiz software (Votingo, Center for Digital Systems (CeDiS), FU Berlin) that was developed from the open‐source web app “Pingo” (https://blogs.upb.de/pingo/opensource/).

### 2.3 Compounding Course

The compounding course is a mandatory course for fourth‐year veterinary students (7th semester) during which they are allocated to different groups to learn the compounding of medications, labeling, and price calculation for dispensing drugs. The course setup changed significantly over the described period (see Table 1): In 2019, each student attended one “Introduction to Compounding” class and then four courses of 1.5 h each, preparing different drug formulations (powder, solution, ointments). In 2020, social distancing regulations made in‐person teaching almost impossible, and students attended the “Introduction to Compounding” online. They only practiced two recipes (powder and solution) in person, and everything else was taught by specifically pre‐recorded video tutorials of compounding (Figure 1). In 2021, two in‐person classes were taught before teaching again moved to video tutorials because of social distancing recommendations. When we returned to the classroom in 2022, we kept the introduction online, as well as the previously produced videos to support self‐study. We then shortened the course to three in‐person exercises because the new European Regulation (EU) 2019/6 (2022b) restricted the legal scenarios for in‐practice drug preparation: According to Article 88 manufacturing of veterinary medicinal products requires a marketing authorization, however the EU member states can provide an exemption for veterinarians. An exemption is granted for the following processes: preparation, dividing up, changes in packaging or presentation of veterinary medicinal products, when these are carried out solely for retail directly to the public in accordance with Articles 103 and 104. In addition, the Articles 112–114 determine that compounding is only allowed when neither an authorized veterinary medicinal product for the same or another species nor a medicinal product for human use is available within the member state or another EU state. Consequently, extemporaneous preparation of veterinary medicinal products without marketing authorization is limited to simple tasks, for example, diluting approved veterinary any drug preparation medicinal products.

**FIGURE 1:**
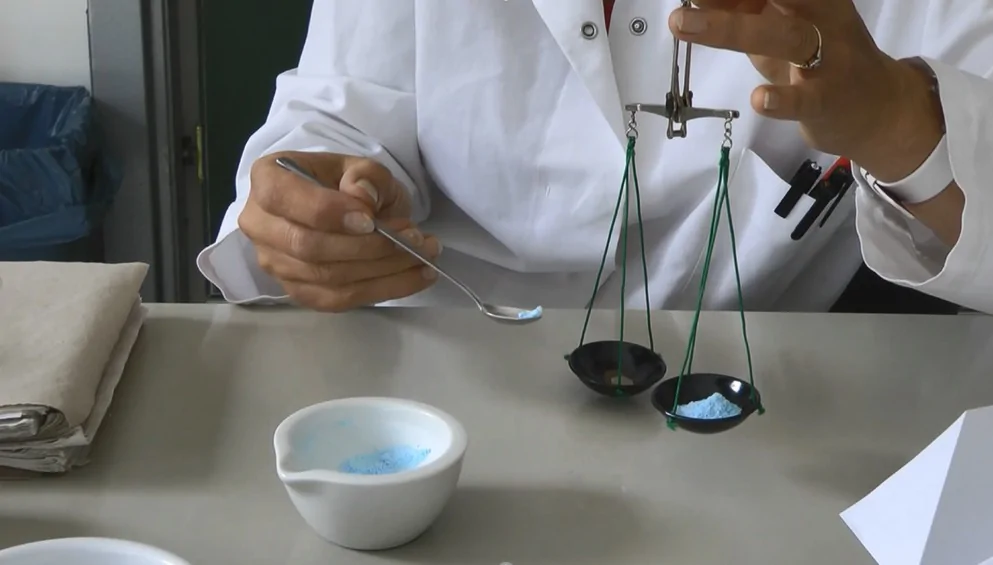
Screenshot from one of the compounding videos on how to prepare different drug formulations. This video teaches how to prepare a powder, the use of manual balances, and the use of mortar and pestle.

As a result of reducing hours in compounding, the hours for practicing prescriptions were increased. We kept the same schedule for 2023 and introduced an additional quiz with which students could earn points toward their practical exam. We used the online platform Kahoot!, a Norwegian online game‐based learning platform, to play live quizzes with the students (KAHOOT! 2024). The questions were created by us based on the content of the instructional videos, were mainly multiple‐choice, or true or false questions with a time limit, and some incorporated images. An example question was “What percentage of a solvens is presented to solve the solvendum?” Questions were displayed via a shared screen and the students selected the answers individually on their own devices. Points were awarded based on accuracy and speed. Each session consisted of four questions, and a leaderboard displayed the winner at the end, adding a competitive element to the quiz.

### 2.4 Prescription Seminar

As noted above, Articles 112–114 of the new EU regulation (2022b) determine the cascade of off‐label use when an authorized veterinary medicinal product for the relevant indication and species is not available. The cascade distinguishes between non‐food‐producing and terrestrial and aquatic food‐producing animals. In all cases, the prescribing veterinarian may exceptionally treat animals with another EU‐authorized veterinary medicinal product. For food‐producing animals, this must be a product authorized for use in food‐producing animals, unless there is none available. In this case, veterinarians can use a veterinary medicinal product for non‐food‐producing animals; however, they do have to verify that the active ingredient is listed in Table 1 of the EU regulation 37/2010 (2009a). Table 2 of this regulation contains forbidden substances for food‐producing animals such as chloramphenicol or metronidazole. There are additional regulations to be aware of in different member states; in Germany, veterinarians also have to adhere to the national Regulation on substances with pharmacological effects (PharmStoffV 2009b), which, for example, forbids the use of specific hormones in food‐producing animals. If no veterinary medicinal product exists, medicinal products for human use can be prescribed. If this option is not available either, a veterinarian may compound a medicinal product or write a prescription for a pharmacy to compound one. As a last resort, veterinary medicinal products for the same indication and species can be imported from third countries. In any case, the withdrawal periods have to be adjusted according to Article 115 (2022b). Students were trained to prescribe veterinary drugs and how to use the above‐mentioned cascades in case‐based learning. After learning the basics of the legislation on prescribing and off‐label use, they were allowed to use a database to find appropriate medications. For VMPs and controlled substances with approval in Germany, they could use VETIDATA, an online veterinary information service for drug use, toxicology, and drug law provided by the Institute of Pharmacology, Pharmacy and Toxicology of the School of Veterinary Medicine, Universität Leipzig (VETIDATA 2024). This platform also includes a withdrawal period calculation tool for off‐label use. We also utilized the Veterinary Medicines Information website, the public interface of the Union Product Database (UPD) to find VMPs with approval in other EU member states (UPD 2024). While other EU member countries have already implemented electronic prescriptions in veterinary medicine, veterinarians are still prescribing on paper in Germany.

In 2019, one in‐person seminar on how to prescribe was held, and some cases were presented. During the pandemic in 2020, this seminar was taught online and we added a second meeting to discuss the cases. From 2021 onwards, we kept the seminar as an online module and introduced a cloud‐based document (Google Docs) to practice prescriptions (Table 1) according to EU and national regulations and controlled substances according to national law (Betäubungsmittelgesetz, BtMG (1981)) for different indications. Students were allocated to groups and worked on a specific clinical case, for example, lameness in a horse, in a virtual room. Results were shared with all students. Students commented anonymously in the document. The course administrator corrected the prescriptions and helped clarify the remaining questions. Students could post questions anonymously in the document until shortly before the exam and faculty answered (Figure 2).

**FIGURE 2:**
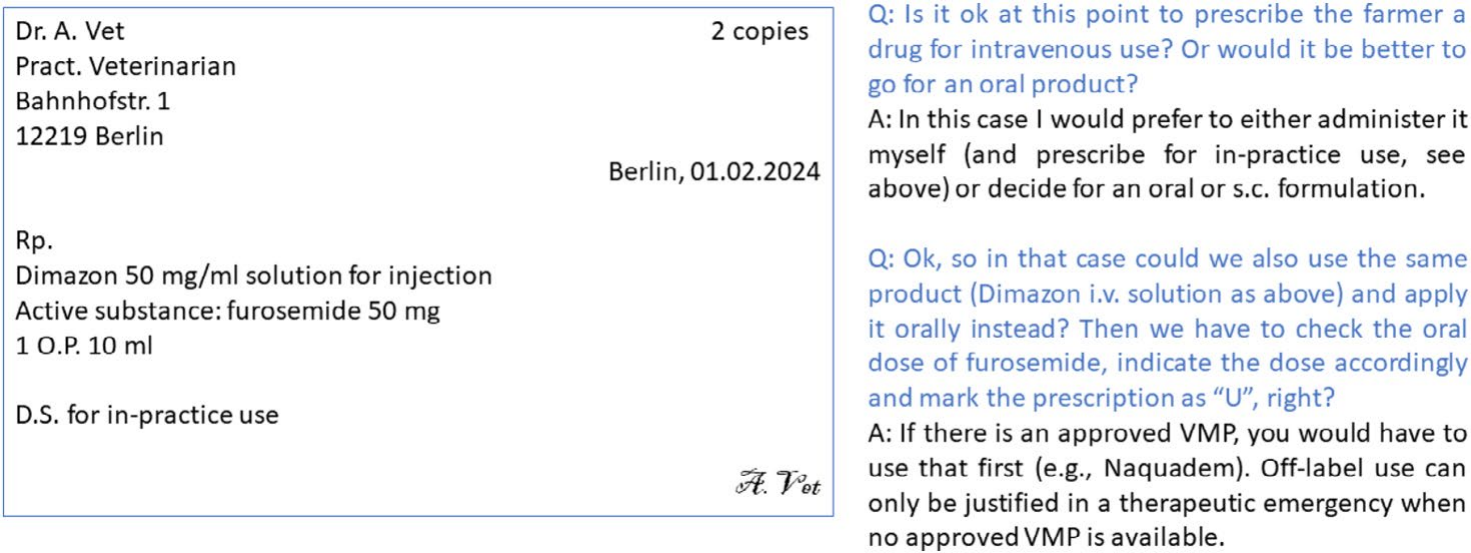
Screenshot from cloud‐based prescription practice document. Shown is a prescription for the task: Prescribe an edema flushing agent for one dairy cow (500 kg). The students decided to prescribe an i.v. injectable drug (Dimazon) to be used by the prescribing vet (in‐practice use) and added some questions below about alternative administration routes. Translated using Deepl.D.S., da signa; O.P., original package; Rp., recipe; U, Umwidmung, off‐label use; VMP, veterinary medicinal product.

### 2.5 Exams and Performance

The basic requirement for participation in the aforementioned classes within the subject “Pharmaceutical and narcotics law” is that students have already participated in basic and special pharmacology in the third year of veterinary school. In order to qualify for the exams that are described in the following, they have to have passed their pharmacology exam. The “Pharmaceutical and narcotics law” exam consisted of three parts. First, a practical compounding exam was performed: Students were asked to prepare two drug formulations, including labeling and price calculation, for example, a thiamazole ointment for transdermal application in cats. This exam counted 20% towards the final grade, and students could reach up to 40 points. Notably, during exams we decided to only ask for recipes that had been taught in person during the specific year, meaning that in 2020 and 2021, the amount of exam recipes was reduced significantly. In 2023, the students could earn extra points towards their compounding exam by winning the quizzes.

Second, two prescriptions for veterinary indications had to be prepared, for example, a diuretic for edema in cattle as presented in Figure 2. During the exam, students had access to the German database VETIDATA. This exam also counted 20% towards the final grade, and up to 40 points were obtainable.

Finally, the remaining 60% of the grade was evaluated during an oral exam, consisting of another oral prescription for a specific veterinary indication and a question on legislation, for example, “what are withdrawal periods and how are they determined?”. The students could earn up to 120 points in the oral section of the exam. In total, in the described three exam parts, students were graded with numbers from 1 (equals A, 170–200 points) to 5 (equals F, fail, less than 100 points).

In 2019–2023, between 135 and 161 students took the practical tests and the general exam, of which between 8.8% and 17.6% were male. We evaluated their performance in the individual practical tests, as well as in the general exam.

### 2.6 Evaluation

The students evaluated the class in 2020 (*n* = 64), 2021 (*n* = 54), and 2022 (*n* = 97). Not all students evaluated and answered every question; all evaluations were performed voluntarily within our regular teaching evaluation cycle. In 2020 and 2021, the focus of the questionnaire was set on digital learning. The results of the evaluation section on the use of digital media and independent learning were translated from German to English using DeepL for this manuscript and are presented in the results.

### 2.7 Statistics

No data were excluded. Data were tested for normality and considering the not‐normal distribution, the Kruskal Wallis test, followed by a Dunn's test as post hoc have been applied. *p* < 0.05 was considered a significant value.

## 3 Results

### 3.1 Class Formats

The lecture itself did not change considerably over the evaluated time period, even though it was alternated from in‐person to online and back to the classroom. Online formats, in general, led to higher attendance rates; however, we did not collect data to support this finding. The students repeatedly praised the quizzes in their evaluation as helpful in assessing their knowledge during the classes. Additionally, they liked the lectures and perspectives from external referees that were designed to show the relevance of the subject and potential veterinary professional careers with a link to drug legislation.

The compounding course and prescription seminar implemented the biggest changes since social distancing regulations forced us to transition to online teaching rather abruptly. As we saw positive student evaluations and better exam outcomes after introducing blended and case‐based learning, we decided to keep some of the newly implemented tools after returning to the classroom, such as the video tutorials in compounding and the online meetings to practice prescriptions within a cloud‐based document.

### 3.2 Student Evaluations

In 2020 and 2021, at the height of online teaching, digital use of media and offers for independent learning were specifically assessed in student evaluations. About 30%–40% of students took part in the voluntary evaluations. In both years, most students evaluated the presented digital materials as very helpful, as well as quizzes and the chat function during online sessions, which most likely included the cloud‐based document with questions and answers for prescriptions (Figure 3). Similarly, most students evaluated those tools as very helpful in independent learning (Figure 4).

**FIGURE 3:**
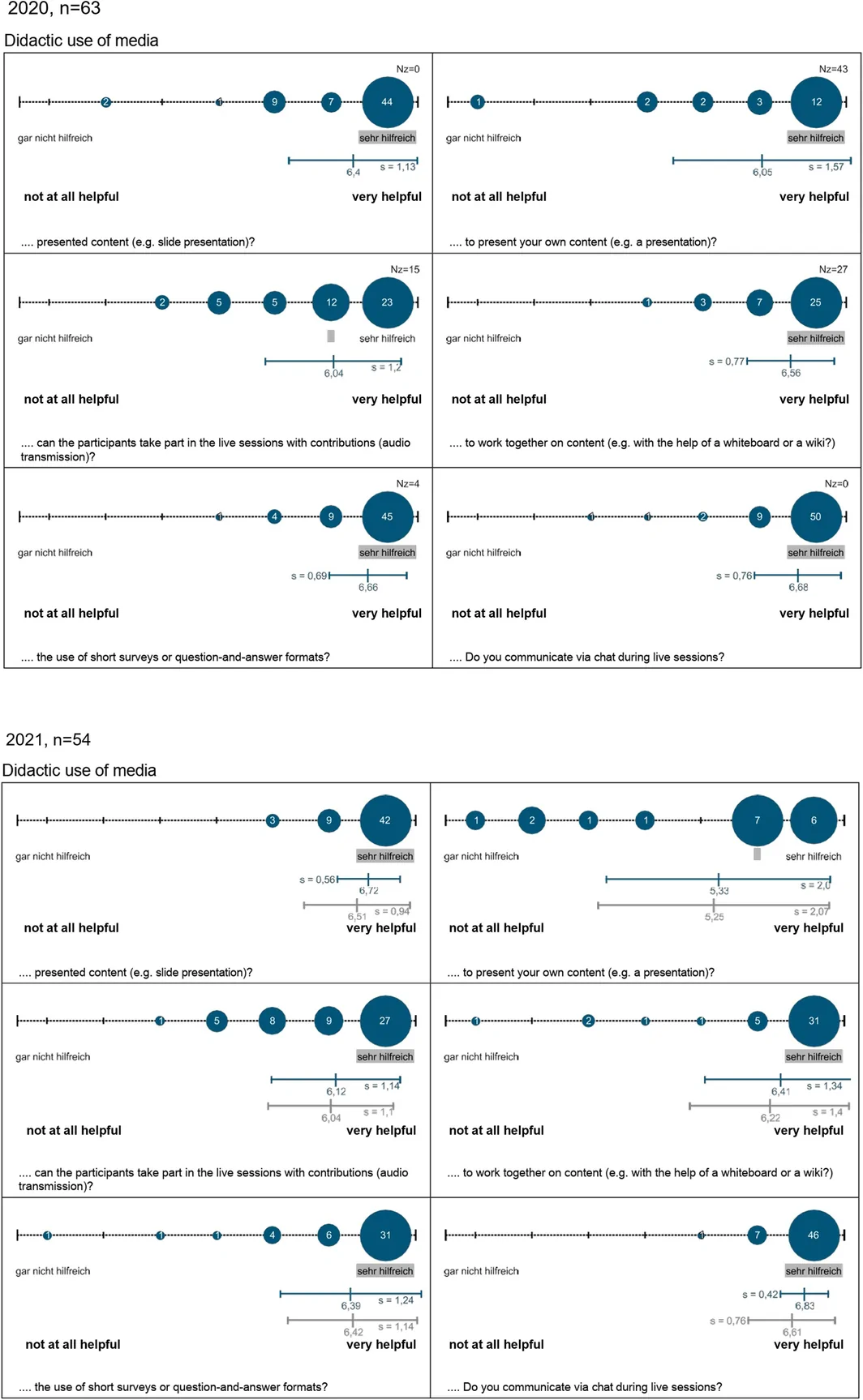
Student evaluations in 2020 and 2021 regarding the didactic use of digital media. Participation in the evaluations was voluntary, and about 30%–40% of students evaluated our class.

**FIGURE 4:**
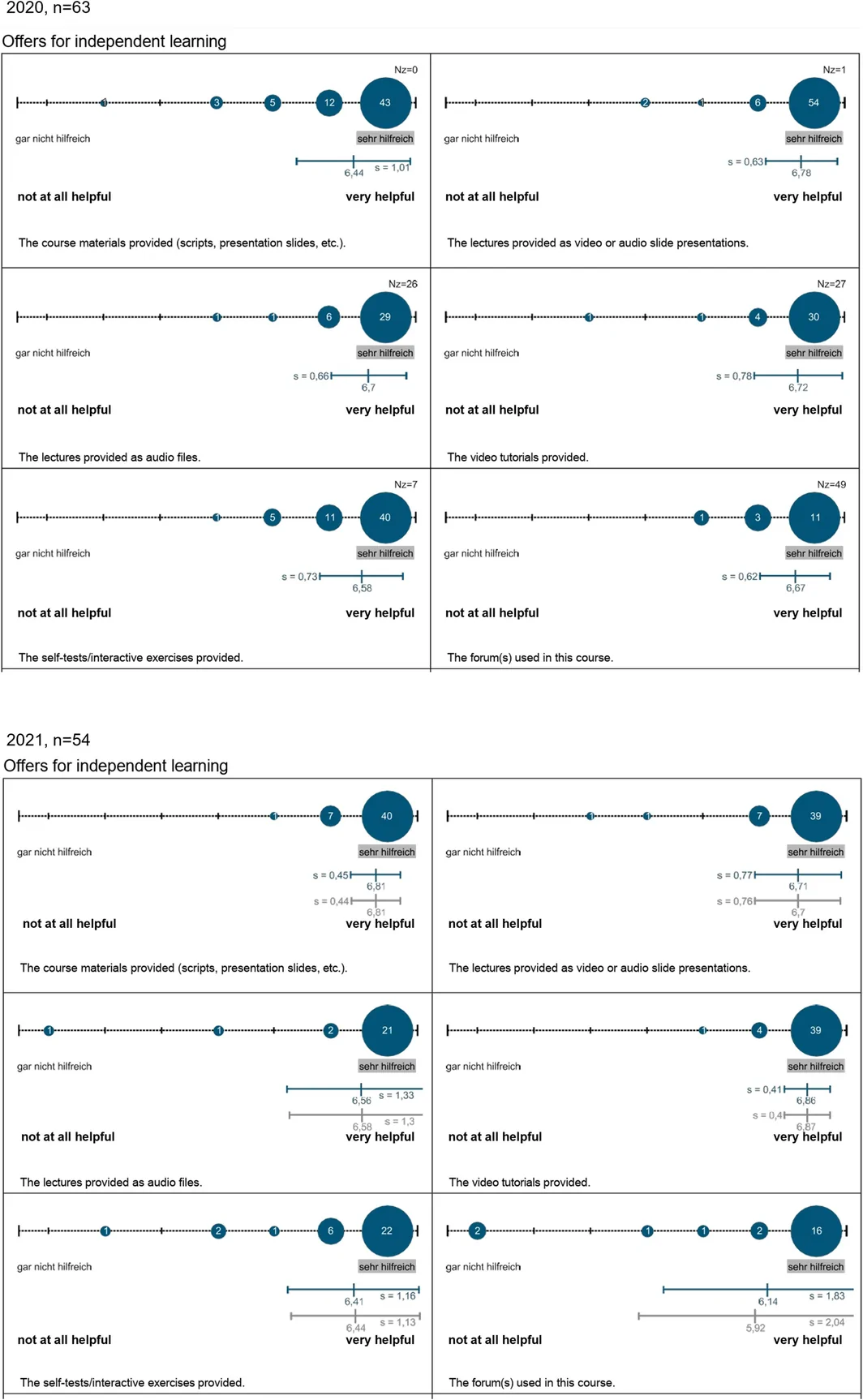
Student evaluations in 2020 and 2021 regarding offers for independent learning. Participation in the evaluations was voluntary, and about 30%–40% of students evaluated our class.

### 3.3 Student Performance

We saw improved grades across time in all exam parts, independent of in‐person or remote teaching.

During the pandemic years 2020 and 2021, we found that our blended‐learning approach with online video tutorials significantly improved the students' performance in the compounding test compared to 2019. However, as mentioned earlier, there was less material to study. After the return to the classroom, achieved points dropped back to pre‐pandemic levels in 2022 but increased again in 2023 after the introduction of extra points through quizzes.

Doubling the time in the prescription seminar to practice more did not lead to improved outcomes in 2020. However, the students achieved more points in the practical test for prescriptions once we introduced the cloud‐based document for case‐based learning in 2021.

The overall grades in pharmaceutical and narcotics law have improved significantly since the introduction of novel blended‐ and case‐based learning approaches in 2020 and have remained on a high level since then (Figure 5).

**FIGURE 5:**
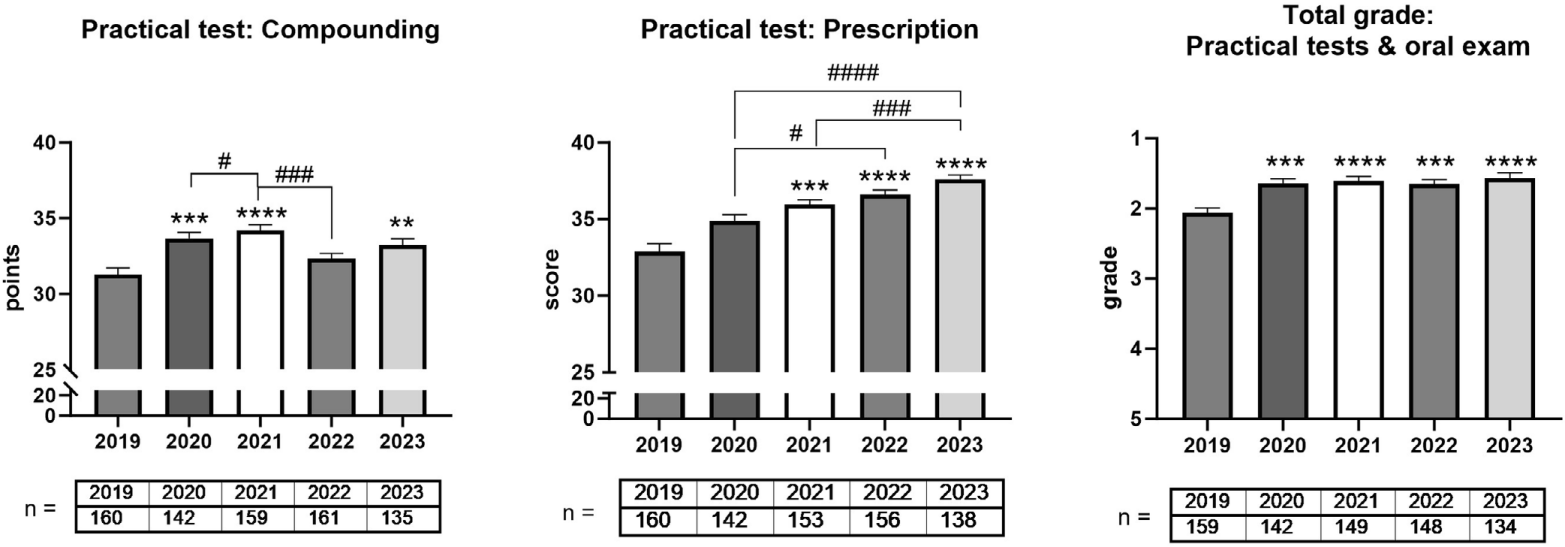
Statistical evaluation of student performance between 2019 and 2023 in the three parts of the exam. All data are mean + SEM, Kruskal Wallis test, post hoc Dunn's. *p* < 0.05 is considered significant. Asterisks show significant differences versus 2019, **p < 0.01, ***p < 0.001, ****p < 0.0001. Rhomb indicates a significant difference between the linked groups, #*p* < 0.05, ###*p* < 0.001, ####*p* < 0.0001.

## 4 Discussion

Teaching pharmaceutical and narcotics law presents the challenge of conveying complex federal and European legislation to students. Additionally, national regulations advocate for practical exercises in prescribing and compounding drugs. Particularly the latter seems specific to Germany. According to the literature, veterinary compounding is primarily taught to pharmacy students, often as elective courses, rather than to veterinary medicine students (McDowell et al. 2017; Fredrickson and Parry 2022; Lust 2004). This approach aligns with the regulatory framework, which restricts compounding activities in veterinary practice to a few specific tasks, such as dilutions. Conversely, pharmacies routinely compound specific veterinary prescriptions for off‐label drug use (Lust 2004). However, despite these limitations, a compounding course is utilized to acquaint veterinary medicine students with fundamental compounding principles and to illustrate differences between, for example, emulsions and suspension ointments. Moreover, mastering everyday skills like precise dilutions and tablet pulverization proves beneficial for their professional development.

Contrary to compounding, prescribing remains an essential first‐day skill for veterinarians worldwide, which has been confirmed by an international consortium of veterinary pharmacology educators recently (Werners and Fajt 2021). They aimed to specifically identify the indispensable day‐1 competences of veterinary graduates in basic and clinical pharmacology. While they found the time allocated for pharmacology education varies across countries, they concluded that one out of eight of the most important skills a graduate should master is choosing a drug and a dose for their patients (Werners and Fajt 2021). Another one of those eight skills was knowing about controlled substances and the associated regulations, including the specifics of controlled substance prescriptions. As pointed out earlier, prescribing errors account for the majority of preventable maltreatment in human patients and most likely also in animals (Hodkinson et al. 2020; Hepworth‐Warren et al. 2024), underlining the importance of drug selection, dose calculations, and formally correct prescribing. In some countries, including Germany, most veterinarians can prescribe out of their in‐house pharmacy; thus, trained veterinary professionals dispense the drugs. However, in case the drug is not used very frequently or the vet has to prescribe a human medication for specific indications and patients (off‐label use), they might write a prescription that will be filled at a community pharmacy. This is common practice across many other countries, where veterinarians do not dispense drugs. It has been reported that especially missing information on prescriptions prevented pharmacists from verifying the appropriateness of the prescribed drug and dose, leading to medication errors (McDaniel et al. 2022). It could be argued that electronic prescriptions would lead to a reduced number of mistakes because usually such systems and forms alert the user if information is missing or does not follow legal regulations. Some countries have already implemented electronic prescriptions in veterinary medicine. The European Commission is working to implement a model format for veterinary prescriptions, which will be available electronically as well. However, in our opinion, training on how to write a hard copy prescription including legally and clinically required information should remain part of veterinary education, as not all practices are digitally up‐to‐date. Nevertheless, electronic tools such as drug databases are and will remain essential aids in selecting drugs and prescribing correctly.

Large international studies with medical students have shown that they were not satisfied with their pharmacology education, and that they felt not adequately prepared to write prescriptions after graduation (Brinkman et al. 2017). The students estimated in questionnaires that they mostly studied pharmacology in self‐directed learning (more than 200 h), followed by traditional lectures and seminars (78 h). The minority of time was spent directly with a tutor (22 h), or with e‐learning approaches (13 h) (Brinkman et al. 2017). When problem‐based learning was introduced, learning outcomes, prescription skills, and student satisfaction significantly improved (Brinkman et al. 2021). This is in line with our finding that simply increasing time for self‐study in prescribing did not lead to better performances in the final exam, while introducing a cloud‐based document with case‐based learning did significantly improve outcomes.

In our hands, pandemic online teaching did not influence the outcomes in practical or theoretical knowledge regarding the students' grade performances. Similarly, the same overall outcome was seen in two cohorts of medical students when online‐only pandemic teaching (cohort 1) was compared to a blended learning environment (cohort 2), even though the blended learning cohort showed higher scores in deep and strategic learning and motivation during the semester (Avraam et al. 2025). In our study, however, novel tools for blended and case‐based learning did improve outcomes. The pros and cons of online teaching during the COVID‐19 Pandemic have already been the subject of previous studies (Simons et al. 2023) and one of the conclusions was that “flexible time management and a shift toward student‐centered learning using strategies such as flipped classrooms may be beneficial” (Parkes and Barrs 2021). Several other universities with degree programs in veterinary medicine have implemented case‐based and e‐learning as a result of social distancing requirements, for example, case‐based and blended learning were evaluated positively by veterinary students in the subject of public health, which usually heavily relied on theoretical lectures in our school (Duckwitz et al. 2022). Students were more content and also assessed their learning outcomes positively. Various preclinical and clinical teaching formats were successfully changed into blended learning concepts internationally (Kelly et al. 2021; Fairs et al. 2023; Green and Whitburn 2016). Other critical skills such as communication training have also been transformed into simulation‐based classes (Heise et al. 2024; Bahramsoltani et al. 2023). On the other hand, focusing solely on online teaching is considered less favorable by students: A systematic review of face‐to‐face versus online teaching in veterinary anatomy revealed that academic performance did not differ, while students were more satisfied with face‐to‐face teaching (Abualadas and Xu 2023).

The benefit of flipped classroom settings allows the students to familiarize themselves with the basics at home before attending the practical course. Flipped classroom scenarios have been reported to increase motivation and performance in veterinary students (Dooley et al. 2018). In our classes this setting allowed them to participate and actively apply their knowledge during in‐person teaching. In order to enhance self‐study, we offered several video tutorials, in which the various recipes and necessary equipment were explained and demonstrated. We kept using several electronic resources after returning to the classroom. There have been several reports about flipped classroom teaching in different courses in veterinary medicine, mostly showing a benefit for the students in their perception and also in the instructor's perception (Dooley et al. 2018; Matthew et al. 2019; Gómez et al. 2023), although a flipped classroom does not necessarily lead to improved student performance in all cases (Moffett and Mill 2014). In fact, we saw a transient decline in performance in compounding. The students might have had some difficulties returning to in‐person learning in the beginning; however, the long‐term outcome of online and blended learning concepts was positive. We have not evaluated any reasons for lower performance in compounding in 2022. Nevertheless, it is important to note that academic grades do not necessarily reflect “Day One” competencies. Many students adopt surface or strategic learning approaches, which may limit long‐term retention and practical application of knowledge. Avraam et al. (2025) have recently shown that deep learning—meaning that students focus on comprehension rather than memorizing information, does not lead to better academic outcomes in medical students. Interestingly, they also showed that deep learning decreased in cohorts of first year medical students over the academic year, despite the use of problem‐based learning approaches (Avraam et al. 2025). This decline was mainly observed among white male students, especially those who were older, and held biomedical sciences degrees. Since our student body is very uniform and largely consists of white, female students, who are younger and do not have any previous undergraduate degrees, one could speculate that motivation and deep learning could be higher, however this was not assessed by us.

### 4.1 Limitations of This Study

Some limitations of this study restrict the interpretation of our results and thus should be discussed. First of all, since this is a single institution study, results should be evaluated carefully and can be different in other educational settings. Furthermore, the comparability across all assessed years is not ideal because the compounding exams during the pandemic were “easier” (consisting of only two preparations instead of four). In addition, we realized that the oral pharmacology exams that were held in parallel for a different cohort of students during the pandemic showed also a better outcome compared to pre‐pandemic evaluations. One interpretation was that students did not have access to alternative activities like sports or social events during the lockdown and might have focused more on learning and preparing for exams, however, this is speculative. Furthermore, oral exams made up 60% of the final grade. While we feel that oral exams are an ideal tool to practice and assess communication skills in students while discussing complex medical and in our case pharmacology content, we understand that this is discussed controversially since performance, difficulty of exam questions, and finally grading are not as objective as written tests with the same questions for every student. However, we have established an extensive catalog of questions and exemplary answers for the respective grades that are regularly discussed among examiners to calibrate our assessments before and again after exams, resulting in a low variability between examiners, for example, in the last exam season all five examiners did not differ in their mean grades (data not shown).

Nonetheless, the consistent overall increase in total grades across all years (2020–2023) compared to the pre‐pandemic period (2019) suggests that the modifications made to the different modules, for example, lectures (including quizzes), compounding course (utilizing videos on compounding), and prescription seminar (incorporating cases, meetings, and cloud‐based documents) have been advantageous for the students. Lastly, it is worth noting that the compounding course was honored with the School of Veterinary Medicine's Teaching Prize in 2023 as the best practical course during clinical education, as voted by the students.

## 5 Conclusion

We hope to provide evidence for the benefits of incorporating active learning strategies in compounding and prescribing education for veterinary students. We recommend educators to try different methods and media to assess what works best for them and their students. Furthermore, we advise to perform frequent student evaluations and not to just base the success of novel tools on student grade performances. While we could show that activities such as blended‐ and case‐based learning can improve student performance and grades in practical and theoretical exams, independent of in‐person or remote teaching, further studies should investigate engagement and motivation to understand pharmacology and regulatory concepts from the student's perspective.

## Author Contributions

All three authors have made substantial contributions to conception and design, or acquisition of data, or analysis and interpretation of data; all authors have been involved in drafting the manuscript or revising it critically for important intellectual content; all authors have given final approval of the version to be published, and have agreed to be accountable for all aspects of the work in ensuring that questions related to the accuracy or integrity of any part of the work are appropriately investigated and resolved.

## Funding

The presented research was performed during the regular teaching obligations; no extra funding besides the teaching budget of our institute was acquired.

## Ethics Statement

The authors confirm that the ethical policies of the journal, as noted on the journal's author guidelines page, have been adhered to. No ethical approval was required as no animals were used.

## Conflicts of Interest

The authors declare no conflicts of interest.

## Data Availability

The data that support the findings of this study are available from the corresponding author upon reasonable request.

## References

[jvp70062-bib-0001] Abualadas, H. M. , and L. Xu . 2023. “Achievement of Learning Outcomes in Non‐Traditional (Online) Versus Traditional (Face‐To‐Face) Anatomy Teaching in Medical Schools: A Mixed Method Systematic Review.” Clinical Anatomy 36: 50–76. 10.1002/ca.23942 PMC10087909 35969356

[jvp70062-bib-0002] Avraam, D. , I. Televantou , A. P. Albert , et al. 2025. “Exploring the Relationship Between Learning Approaches and Problem‐Based Learning: Insights From a Longitudinal Study in Medical Students.” BMC Medical Education 25: 619. 10.1186/s12909-025-07171-1 PMC12032700 40287686

[jvp70062-bib-0003] Bahramsoltani, M. , S. Bröer , S. Langforth , et al. 2023. “Outcome of Communication Training in Veterinary Studies: Influence on the Perception of the Relevance of Veterinary Competencies and Self‐Assessment of Communication Skills.” Animals (Basel) 13, no. 9: 1516. 10.3390/ani13091516 PMC10177072 37174553

[jvp70062-bib-0004] Brinkman, D. J. , T. Monteiro , E. C. Monteiro , M. C. Richir , M. A. VAN Agtmael , and J. Tichelaar . 2021. “Switching From a Traditional Undergraduate Programme in (Clinical) Pharmacology and Therapeutics to a Problem‐Based Learning Programme.” European Journal of Clinical Pharmacology 77: 421–429. 10.1007/s00228-020-03027-3 PMC7867513 33098019

[jvp70062-bib-0005] Brinkman, D. J. , J. Tichelaar , T. Schutte , et al. 2017. “Essential Competencies in Prescribing: A First European Cross‐Sectional Study Among 895 Final‐Year Medical Students.” Clinical Pharmacology and Therapeutics 101: 281–289. 10.1002/cpt.521 PMC5260337 27648725

[jvp70062-bib-0006] Dooley, L. M. , S. Frankland , E. Boller , and E. Tudor . 2018. “Implementing the Flipped Classroom in a Veterinary Pre‐Clinical Science Course: Student Engagement, Performance, and Satisfaction.” Journal of Veterinary Medical Education 45: 195–203. 10.3138/jvme.1116-173r 29393765

[jvp70062-bib-0034] Dornan, T. , W. Ashcroft , H. Heathfield , et al. 2009. “An in Depth Investigation into Causes of Prescribing Errors by Foundation Trainees in Relation to their Medical Education – EQUIP Study.” https://www.gmc‐uk.org/cdn/documents/final‐report‐prevalence‐and‐causes‐of‐prescribing‐errors_pdf‐28935150.pdf.

[jvp70062-bib-0007] Duckwitz, V. , L. Vogt , C. Hautzinger , et al. 2022. “Teaching Outbreak Investigations With an Interactive Blended Learning Approach.” Journal of Veterinary Medical Education 49: 312–322. 10.3138/jvme-2020-0077 34129432

[jvp70062-bib-0008] EAEVE . 2019. List of Subjects and Day One Competences Accessed June 3, 2024. https://www.eaeve.org/fileadmin/downloads/eccvt/List_of_subjects_and_Day_One_Competences_approved_on_17_January_2019.pdf.

[jvp70062-bib-0009] Fairs, J. , A. Conan , K. Yvorchuk‐St Jean , et al. 2023. “The Evaluation of a High‐Fidelity Simulation Model and Video Instruction Used to Teach Canine Dental Skills to Pre‐Clinical Veterinary Students.” Veterinary Sciences 10, no. 8: 526. 10.3390/vetsci10080526 PMC10458249 37624313

[jvp70062-bib-0010] Fredrickson, M. E. , and V. Parry . 2022. “Purr‐Fecting Skills: Design of a Piloted Veterinary Pharmacy Elective Course With a Compounding Focus.” Currents in Pharmacy Teaching & Learning 14: 1040–1045. 10.1016/j.cptl.2022.07.013 36055694

[jvp70062-bib-0011] Gómez, O. , M. García‐Manzanares , D. Chicharro , et al. 2023. “Application of Blended Learning to Veterinary Gross Anatomy Practical Sessions: Students' Perceptions of Their Learning Experience and Academic Outcomes.” Animals (Basel) 13, no. 10: 1666. 10.3390/ani13101666 PMC10215638 37238097

[jvp70062-bib-0012] Green, R. A. , and L. Y. Whitburn . 2016. “Impact of Introduction of Blended Learning in Gross Anatomy on Student Outcomes.” Anatomical Sciences Education 9: 422–430. 10.1002/ase.1602 26929149

[jvp70062-bib-0013] Heise, S. A. C. , S. Wissing , V. Nerschbach , E. Preussing , A. Tipold , and C. Kleinsorgen . 2024. “Evaluation of an Interprofessional Blended Learning Course Focusing on Communication Within Veterinary Teams.” Animals (Basel) 14, no. 5: 729. 10.3390/ani14050729 PMC10931423 38473115

[jvp70062-bib-0014] Hepworth‐Warren, K. L. , E. Maynard‐Swift , T. Prange , et al. 2024. “Error Reporting in a Large Animal Veterinary Teaching Hospital Identifies Medication Errors Occur Most Often in the Prescribing Phase of Therapy.” Journal of the American Veterinary Medical Association 262: 1–7. 10.2460/javma.23.10.0556 38134457

[jvp70062-bib-0015] Hodkinson, A. , N. Tyler , D. M. Ashcroft , et al. 2020. “Preventable Medication Harm Across Health Care Settings: A Systematic Review and Meta‐Analysis.” BMC Medicine 18: 313. 10.1186/s12916-020-01774-9 PMC7646069 33153451

[jvp70062-bib-0016] KAHOOT! . 2024. Global Learning and Engagement Platform Accessed August 11, 2024. https://kahoot.com/.

[jvp70062-bib-0017] Kelly, R. F. , M. Mihm‐Carmichael , and J. A. Hammond . 2021. “Students' Engagement in and Perceptions of Blended Learning in a Clinical Module in a Veterinary Degree Program.” Journal of Veterinary Medical Education 48: 181–195. 10.3138/jvme.2019-0018 31873057

[jvp70062-bib-0018] Lust, E. 2004. “Compounding for Animal Patients: Contemporary Issues.” Journal of the American Pharmacists Association 44: 375–384; quiz 384–386. 10.1331/154434504323064011 15191248

[jvp70062-bib-0019] Matthew, S. M. , R. M. Schoenfeld‐Tacher , J. A. Danielson , and S. M. Warman . 2019. “Flipped Classroom Use in Veterinary Education: A Multinational Survey of Faculty Experiences.” Journal of Veterinary Medical Education 46: 97–107. 10.3138/jvme.0517-058r1 30418806

[jvp70062-bib-0020] McDaniel, M. , E. P. C. Glazier , N. P. C. Truong , et al. 2022. “Veterinary Prescription Errors in a Community Pharmacy Setting: A Retrospective Review.” Journal of the American Pharmacists Association 62: 512–518. 10.1016/j.japh.2021.11.007 34872858

[jvp70062-bib-0021] McDowell, A. , R. Beard , A. Brightmore , et al. 2017. “Veterinary Pharmaceutics: An Opportunity for Interprofessional Education in New Zealand?” Pharmaceutics 9, no. 3: 25. 10.3390/pharmaceutics9030025 PMC5620566 28933730

[jvp70062-bib-0022] Moffett, J. , and A. C. Mill . 2014. “Evaluation of the Flipped Classroom Approach in a Veterinary Professional Skills Course.” Advances in Medical Education and Practice 5: 415–425. 10.2147/AMEP.S70160 PMC4235505 25419164

[jvp70062-bib-0023] Parkes, R. S. V. , and V. R. D. Barrs . 2021. “Interaction Identified as Both a Challenge and a Benefit in a Rapid Switch to Online Teaching During the COVID‐19 Pandemic.” Journal of Veterinary Medical Education 48: 629–635. 10.3138/jvme-2020-0063 33493099

[jvp70062-bib-0024] Regulation . 1981. Gesetz über den Verkehr Mit Betäubungsmitteln (Betäubungsmittelgesetz—BtMG). Accessed June 3, 2024. https://www.gesetze‐im‐internet.de/btmg_1981/BJNR106810981.html.

[jvp70062-bib-0025] Regulation . 2006. Verordnung Zur Approbation von Tierärztinnen Und Tierärzten (TAppV). Accessed June 3, 2024. https://www.gesetze‐im‐internet.de/tappv/BJNR182700006.html.

[jvp70062-bib-0026] Regulation . 2009a. Regulation (EU) no. 37/2010 on Pharmacologically Active Substances and Their Classification Regarding Maximum Residue Limits in Foodstuffs of Animal Origin. European Union. Assessed February 2, 2026. https://eur‐lex.europa.eu/eli/reg/2010/37(1)/oj/eng.

[jvp70062-bib-0029] Regulation . 2009b. Verordnung über Stoffe mit Pharmakologischer Wirkung. Accessed February 2, 2026. https://www.gesetze‐im‐internet.de/pharmstv/BJNR014790977.html.

[jvp70062-bib-0027] Regulation . 2022a. Gesetz Über Den Verkehr Mit Tierarzneimitteln Und Zur Durchführung Unionsrechtlicher Vorschriften Betreffend Tierarzneimittel. Accessed June 3, 2024. https://www.gesetze‐im‐internet.de/tamg/.

[jvp70062-bib-0028] Regulation . 2022b. Regulation (EU) 2019/6 of The European Parliament and of The Council of 11 December 2018 on Veterinary Medicinal Products and Repealing Directive 2001/82/EC. European Union. Accessed June 3, 2024. https://eur‐lex.europa.eu/legal‐content/DE/ALL/?uri=CELEX%3A32019R0006.

[jvp70062-bib-0030] Simons, M. C. , D. Pulliam , and J. A. Hunt . 2023. “The Impact of the COVID‐19 Pandemic on Veterinary Clinical and Professional Skills Teaching Delivery and Assessment Format.” Journal of Veterinary Medical Education 50: 61–76. 10.3138/jvme-2021-0106 35038389

[jvp70062-bib-0031] UPD . 2024. Union Product Database [Online]. European Union Accessed August 11, 2024. https://medicines.health.europa.eu/veterinary/select‐language?destination=/node/210934.

[jvp70062-bib-0032] VETIDATA . 2024. Veterinärmedizinischenr Informationsdienst für Arzneimittelanwendung, Toxikologie Und Arzneimittelrecht [Online]. Institut für Pharmakologie, Pharmazie Und Toxikologie, Veterinärmedizinische Universität Leipzig Accessed June 3, 2024. www.vetidata.de.

[jvp70062-bib-0033] Werners, A. , and V. Fajt . 2021. “What a Veterinary Graduate Should Know About Basic and Clinical Pharmacology: A Delphi Study to Finalize Day‐1 Competencies.” Journal of Veterinary Pharmacology and Therapeutics 44: 568–574. 10.1111/jvp.12920 33078856

